# Supplementary material for: Untangling the tingle: Investigating the association between the Autonomous Sensory Meridian Response (ASMR), neuroticism, and trait & state anxiety
Source: PLoS One. 2022 Feb 2;17(2):e0262668. doi: 10.1371/journal.pone.0262668 (PMC8809551; doi:10.1371/journal.pone.0262668)
Supplement: Attachment — Submitted filename: PLos ONe - response_to_reviewers.docx [file pone.0262668.s001.docx]

Reviewer #1: 

1: The literature is reviewed in a clear and concise manner. As noted by the authors, an early study showed that watching ASMR stimuli can improve participant mood for several hours, suggesting that depressed people derive the greatest benefit from engaging in ASMR (Barratt & Davis, 2015). A recent study demonstrated that sensitivity to ASMR stimuli is correlated with participant anxiety state (Koumura et al., 2021, Q J Exp Psychol), although the authors did not cite this paper. What is the critical difference between anxiety and mood? How are the present and previous findings related? The authors should discuss these issues.

Response: We thank Reviewer 1 for drawing our attention to this paper which was not available at the time of submission of our manuscript and have added this in the development of our arguments (lines 85, 355). Whilst we agree that there are critical differences between mood and anxiety, due to the novelty of ASMR as a focus of empirical research along with our specific research question, we do not feel that detailed discussion is of such relevance to our paper though we welcome future consideration of this.

2: The authors claimed selection bias of participants as a limitation of this study. They excluded 10 participants as ASMR experiencers and included 8 ASMR non-experiencers among ASMR experiencers. Is it possible to derive generalized principles when using such methods? In addition, the authors used only one ASMR stimulus. Indeed, the stimulus consisted of some situations (e.g., brushing, tapping, and scratching), but was rapidly changing. When the authors checked the participant ASMR experiences, I consider the following questions inappropriate: Did this video relax/calm you? or did this video make you feel sleepy? (p. 9) It is better to ask participants directly whether they really experienced a tingling sensation.

Response: On reviewing our text, we can see that greater clarification is required regarding the criteria in the allocation of participants to the ASMR-experiencer or non-experiencer groups. Group allocation was based on participants’ responses to both pre-study and post-study video ASMR experience questionnaires, and specifically based on the location of the origin of the tingles, which had to be either the head and / or neck. Group allocation was not based on responses to the ASMR video experience questionnaire (which we have now renamed *ASMR video engagement* questionnaire as we feel this provides greater distinction between the two measures). This text has now been removed (see line 128-129). We agree that exclusion and inclusion of participants to these groups was also not clear and have provided greater clarity below in response to Reviewer 2’s similar comments. The manuscript has been amended to clarify group allocation (see lines 127-143) and also the elaborated data set which is available from <https://doi.org/10.25398/rd.northumbria.16654846>.

We also agree that the ASMR video engagement questionnaire was not a sufficient measure in general and we have already acknowledged this in our discussion section (lines 450-457) where we emphasised that more robust measures such as the ASMR-15 Inventory (Roberts et al., 2019) or AEQ (Swart et al., 2021) should be incorporated in future research.

3: The authors performed mediation analyses (Fig. 2), but did not show the correlation between predictor and outcome: Group and change in an anxiety state. If the authors do not find a significant correlation between predictor and outcome, then it would be pointless to carry out a mediation analysis. Thus, they should explicitly show the correlation and then discuss effects of mediators on outcomes.

Response: This correlation has been added on lines 315-316.

Reviewer #2: 
1. In the method section it is mentioned that participants were categorized as either ASMR-experiencers or non-experiencers based on whether they had previously viewed ASMR videos and on their answers to the video and ASMR experience questionnaires. Based on the text, however, it is unclear how these questionnaires were used to determined whether someone was an ASMR-experiencer. The distinction between ASMR-experiencers and non-experiencers needs to be explained better. I think it would also be beneficial to attach all the questionnaires to the appendix.

Response: We agree with Reviewer 2’s comments here and similar were raised by Reviewer 1. We have addressed this above in response to R1 and below in response to R2’s 2^nd^ comment, where we outline in detail how participants were allocated to either the ASMR-experiencer or non-experiencer groups. To clarify, the ASMR video experience (now renamed *video engagement*) questionnaire was not used to classify group allocation and this text has been removed.

2. On line 105-107 it is stated that the first research question was to determine whether ASMR-experiencers and non-experiencers differ on neuroticism, trait, and state anxiety, and the second research question was to determine whether exposure to ASMR videos reduces state anxiety in general or whether it is specific to those who experience ASMR. Based on the text in the participants section on pages 6-7 it appears that 38 participants reported to watch ASMR videos and experience sensations in relation to them, however, 10 of those participants were assigned to the non-experiencer group because they did not experience ASMR tingles during the study. To my understanding, these participants self-identified as ASMR-experiencers. I think assigning participants who report to experience ASMR into a non-ASMR group because they did not experience tingles during the experiment is incorrect and contradicts the analyses conducted, particularly those pertaining to the first two research questions. Participants who report to experience ASMR should not be included in the non-experiencer group.

I would like to thank the authors for an interesting manuscript about an interesting topic. If the analyses are redone with the above group adjustments I would be happy to reconsider.

Response: Thank you for your positive comments regarding our manuscript. We have revisited the full data set and identified that the wording of the allocation of the participants to either the ASMR-experiencers or non-experiencers groups was not clearly documented. However, the error here is in the miswording of the manuscript and not in the mis-categorisation of the participants. Participants were assigned to the ASMR-experiencers group on the basis of experiencing ‘true’ ASMR either pre-video, post-study video, or both. ‘True’ ASMR is experienced when the tingles originate from the head and / or neck (e.g. Tihanyi et al., 2018). To clarify, categorisation of the participants in the study was as follows:

A: Participants who experienced true ASMR pre- and / or post-video: n = 19

B: Participants who experienced true ASMR pre-video but not post-video: n = 9

C: Participants who had never previously experienced ASMR but experienced true ASMR post-video: n = 8

D: Participants who had never previously experienced ASMR prior to participating in the study, and post-video by reporting either no tingles, or tingles that did *not* originate from the head and / or neck: n = 28

The participants allocated to the ASMR-experiencers group consisted of participants from categories A, B, and C (n=36). The non-experiencers group were participants in category D (n=28). The manuscript has been amended accordingly to clarify the group allocation (lines 127-143). To ensure that the issue related to wording only and not mis-categorisation of the participants, we re-ran all the analyses on the above group re-allocation and observed exactly the same results as we reported in the originally submitted manuscript. The elaborated data set which includes the breakdown of these four categories has been uploaded to the Northumbria University Figshare repository (<https://doi.org/10.25398/rd.northumbria.16654846>). Relevant comments in the discussion have either been removed or rephrased (see lines 437-448).
