# Supplementary material for: Untangling the tingle: Investigating the association between the Autonomous Sensory Meridian Response (ASMR), neuroticism, and trait & state anxiety
Source: PLoS One. 2022 Feb 2;17(2):e0262668. doi: 10.1371/journal.pone.0262668 (PMC8809551; doi:10.1371/journal.pone.0262668)
Supplement: Attachment — Submitted filename: Plos one_ response_to_R2_2nd revisions.docx [file pone.0262668.s002.docx]

Reviewer #2: I would like to thank the authors for providing a revised version based on the previous comments. However, I find that the revisions made by the authors thus far were insufficient.

1a: My first issue is related to how the authors differentiate 'true' ASMR-experiencers from false-positives:

On line 188-193 it is mentioned that 'qualifying' questions regarding the location of any tingles was used to assess whether participants truly experienced ASMR. It is unclear to me what these questions where. Based on the text, I understand that only the origin of the sensation was used to make this distinction. This can be done with one question, however, the authors mention that several questions were used. I think it would be important to specify exactly what questions were used to make this distinction.

Response:

The participants were asked two questions: a: ‘*Did you experience a tingling sensation?’* to which they responded ‘yes’ or ‘no’. If the response was ‘yes’ they were asked b: *‘Where did these tingles start’* to which they responded accordingly in a text box. This text clarifying this has been included in lines 197-199. This enabled us to identify false positives – these data are available in the accompanying dataset.

1b: Furthermore, if the origin of the ASMR sensations was used as the sole criterion to differentiate true ASMR experience from false positives, I think it is not clear from the manuscript why this is sufficient. On lines 197-199 the authors refer to the Fredborg et al. (2017) study and claim that they used a similar 'qualifying' questions regarding the location of tingles. Upon examining the referred study, however, I do not find any reporting that would imply that a similar method was used. To my understanding, the qualifying questions mentioned in Fredborg et al. (2017) refer to their ASMR checklist, which does not seem to include questions regarding sensation location.

Response:

Thank you for identifying this. We agree that these qualifying questions were not used in the Fredborg et al. (2017) paper and this wording was included in the manuscript in error. We have removed this text.

1c: To further contradict the use of only the origin of the tingling sensations to differentiate true ASMR from false positives, Barrat & Davis (2015), for example, report that only 63% of their participants reported a consistent origin and 41% of those participants reported the head and 29% the shoulders as the origin. In addition, Swart et al. (2021), who are also referred to in the manuscript, report that false positive ASMR-responders appear to differ most from actual ASMR-responders in that their sensations do not seem to emphasize the head as the most prominent origin of their tingles and typically report them as unpleaseant and not calming.

Response:

The paper by Barratt and Davis (2015) states that ASMR is typically characterised by tingles that originate from the head *‘to achieve a tingling, static-like sensation widely reported to spread across the skull and down the back of the neck (Taylor, 2014)*’ and this distinction is reported repeatedly within the ASMR literature. This classification enables researchers to dissociate what we have referred to as ‘true’ ASMR from frisson (which is a different sensation e.g. piloerection) and originates in other parts of the body. We have been clear about this in the manuscript – see lines 190-192. Whilst we disagree with Barratt and Davis’ inclusion of the above mentioned 29% as ASMR-experiencers, theirs was a seminal research paper in the area, however knowledge and understanding has advanced since then. There will no doubt always be a debate when it comes to subjective measures regarding what ‘true’ ASMR experience actually is. However, in light of the comparisons with frisson, we feel the need to dissociate this. By stating that participants can only be allocated to the ASMR-experiencer group if they experienced tingles originating in the head and / or neck enables us to make that distinction and is consistent with definition in much of the literature. Whilst we respectfully appreciate that some researchers are happy to include sensations originating from other areas of the body as experiencing AMSR, in our opinion, to be classified as an ASMR-experiencer, the sensations must originate from the head and / or neck and we feel we have been very clear describing this.

With regard to R2 comments about the Swart et al. (2021) paper, these authors stated that ‘*An individual would be deemed a false positive when they report experiencing something, however that something does not align to the hallmark features of ASMR (e.g., pleasant, calming, head-dominant tingles* (our emphasis)*; henceforth termed False- Positive)*’. In our manuscript we have been clear that if a sensation does not originate in the head and / or neck, these participants will be allocated to the non-ASMR experiencer group (see lines 130-140 which now includes the word ‘False-Positive’ in order to define this as per R2 comment 2 below). Therefore, we feel that R2’s latter comments here actually concur with our definition.
1d: Therefore, I think the manuscript needs to make more clear how 'true' ASMR experience was determined and clearly specify whether and how the method used is supported by the literature, including further discussion of potential limitations (e.g., might mistaking 'true'. ASMR experiencers as false positives be possible?).

Response:

The first point has been addressed. Whilst we would welcome further debate in the literature regarding true ASMR / false positive reporting we do not feel further elaboration is relevant to the focus of this paper. We have directed the readers to relevant literature.

2: My second (somewhat related) issue is with group assignment and how it is explained:

2a: Although revision to the participants section has been made, I still find the way group assignment is explained not clear. Also, the term 'true' ASMR is mentioned for the first time on line 132 but has not been defined in the preceding text. Since it seems to be the case that experiencing 'true' ASMR is used as the main criterion for group assignment, its meaning should first be explained clearly.

Response:

We have incorporated the definitions of ‘true’ ASMR and false positives in lines 130-133, and 141.

2b: Based on the revised text and the authors responses, it is my understanding that the non-ASMR-experiencer group includes participants who do not report to experience ASMR and also participants who report to experience ASMR but whose experience is not considered 'true' ASMR by the authors. I think that the authors need to explain and motivate their choices for group assignment in the manuscript clearer and in more detail.

Response:

We have addressed this in our response to section 1c.

Minor issue:

On lines 411-414, it is written that this is the first study to assess whether participants were ASMR-experiencers post-video as well as pre-video. I do not understand what this means. Previous studies have also used questionnaires to 'confirm' or validate participants' ASMR experiencers by asking participants if they consider themselves ASMR experiencers and whether they experienced ASMR while watching a video. How is the method used here different from such studies?

Response:

We agree that many studies use questionnaires to validate ASMR experience, however this technique is typically to categorise ASMR-group allocation prior to the experimental protocol. Furthermore, post-video screening has resulted in participants being excluded if their responses do not match their group allocation (e.g. Fredborg et al., 2018; 2020). In our study, this was a strategy to capture genuine ASMR experiences. ASMR-naïve participants would self-classify as non-experiencers. Many have never heard of or been exposed to ASMR videos, therefore do not have any context by which to evaluate if they are an experiencer or not. In our sample, 8 self-categorised non-experiencers *did* experience tingles originating in the head and / or neck post-video. Similarly, this technique enabled us to classify 9 true non-experiencers and avoid false-positive reporting. We have identified a paper that has used the same technique thus added this citation (line 139) and removed the wording from lines 411-413.

I still think that the manuscript is interesting and publishing it has value. If group assignment and 'true' ASMR is properly defined and explained by the authors, and further limitations are discussed, I would recommend that it be accepted for publication.

Response:

We would like to thank Reviewer 2 for their thorough comments.
